# AI-Driven Data Analysis for Asthma Risk Prediction

**DOI:** 10.3390/healthcare13070774

**Published:** 2025-03-31

**Authors:** Meng-Han Chen, Guanling Lee, Lun-Ping Hung

**Affiliations:** 1 Department of Computer Science and Information Engineering, National Dong Hua University, Hualien 974301, Taiwan; guanling@gms.ndhu.edu.tw; 2 Department of Information Management, National Taipei University of Nursing and Health Sciences, Taipei 108306, Taiwan

**Keywords:** artificial intelligence (AI), machine learning (ML), asthma risk prediction

## Abstract

**Background:** Asthma is a well-known otolaryngological and immunological disorder that affects patients worldwide. Currently, the primary diagnosis relies on a combination of clinical history, physical examination findings consistent with asthma, and objective evidence of reversible airflow obstruction. However, the diagnostic process can be invasive and time-consuming, which limits clinical efficiency and accessibility. **Objectives:** In this study, an AI-based prediction system was developed, leveraging voice changes caused by respiratory contraction due to asthma to create a machine learning (ML)-based clinical decision support system. **Methods:** A total of 1500 speech samples—comprising high-pitch, normal-pitch, and low-pitch recitations of the phonemes [i, a, u]—were used. Long-Term Average Spectrum (LTAS) and Single-Frequency Filtering Cepstral Coefficients (SFCCs) were extracted as features for classification. Seven machine learning algorithms were employed to assess the feasibility of asthma prediction. **Results:** The Decision Tree, CNN, and LSTM models achieved average accuracies above 0.8, with results of 0.88, 0.80, and 0.84, respectively. Observational results indicate that the Decision Tree model performed best for high-pitch phonemes, whereas the LSTM model outperformed others in normal-pitch and low-pitch phonemes. Additionally, to validate model efficiency and enhance interpretability, feature importance analysis and overall average spectral analysis were applied. **Conclusions:** This study aims to provide medical clinicians with accurate and reliable decision-making support, improving the efficiency of asthma diagnosis through AI-driven acoustic analysis.

## 1. Introduction

Asthma is a common chronic respiratory immunological disorder [1,2]. The primary symptoms include wheezing, coughing, and breathlessness, which may result from respiratory tract hyperresponsiveness and inflammation [3,4,5]. Its incidence is associated with outdoor allergens such as pollen from trees, grass, and weeds, as well as perennial allergens including dust mites, mice, cockroaches, molds, and pet dander. Additionally, nonallergic triggers such as cigarette smoke, secondhand smoke exposure, irritants, and perfumes can also induce asthma symptoms [6]. Proper diagnosis and management of asthma are critical. Multiple biological agents, such as antigen agonists or biomarkers, have recently become available for rapid diagnosis; however, the high cost of these treatments has hindered their widespread adoption. Therefore, this study utilized various machine learning (ML) tools, which are both non-invasive and cost-effective, to facilitate the clinical diagnosis process.

In recent years, the integration of artificial intelligence (AI) techniques in medical diagnosis has significantly improved healthcare systems [7]. AI leverages large clinical datasets to identify abnormalities, often surpassing human performance in many medical fields. In otolaryngology, disease diagnosis often relies on acoustic evidence, CT imaging, or radiology, with tools such as digital otoscopy and tympanometry being commonly used; however, these methods still pose intrusiveness issues. AI algorithms, through ML techniques, systematically analyze acoustic signals and images, enabling more comprehensive disease detection. By applying ML techniques, AI can extract key features and classify them using various deep learning models, allowing doctors to predict diseases with higher accuracy and improving medical diagnostic efficiency.

The application of AI in asthma prediction has grown significantly, with ML tools widely used to classify various features extracted from clinical datasets [8]. However, many ML-based predictions lack transparency and interpretability, making it difficult for doctors to assess the results. To address this issue, explainable AI (XAI) has been introduced. Currently, the most common XAI methods are SHAP and LIME, with SHAP providing both global and local explanations, while LIME only offers local explanations [9]. Therefore, this study utilized SHAP to ensure a more comprehensive explanation of the results.

This study employed seven machine learning algorithms—Decision Tree, Random Forest, Gradient Boosting (GBDT), Support Vector Machine (SVM), Artificial Neural Network (ANN), Convolutional Neural Networks (CNNs), and Long Short-Term Memory (LSTM)—implemented using the scikit-learn package (version 1.1.2) in Python 3.10.12 for asthma identification. A total of 1500 samples of three types of speech materials—high-pitch, normal-pitch, and low-pitch recitations of the phonemes [i, a, u]—were used, sourced from the Saarbruecken Voice Database. Long-Term Average Spectrum (LTAS) and Single-Frequency Filtering Cepstral Coefficients (SFCCs) were extracted as features for classification. To prevent overfitting, 80% of the dataset was allocated for model training, while the remaining 20% was used for testing.

## 2. Related Works

Since traditional examination tools such as otoscopic checks, CT scans, and blood serum biomarker level assessments are invasive, the application of machine learning in asthma prediction has gained popularity [10,11,12,13]. Numerous studies have evaluated its effectiveness, as illustrated by the following examples from previously published research:

Kocsis et al. tested three machine learning models—Support Vector Machines (SVMs), Random Forests, and AdaBoost—for asthma prediction [10]. These models were assessed based on their classification capability in monitoring each patient’s condition. The results indicate that the Random Forest algorithm achieved higher accuracy in predicting asthma control status compared to SVM and AdaBoost classifiers.

The study by Kumar et al. explores childhood asthma prediction using five machine learning models: Linear Regression, K-Nearest Neighbors (KNNs), Decision Trees, Random Forests, and Support Vector Machines (SVMs) [11]. The authors introduce a novel approach that integrates advanced feature selection techniques with supervised machine learning algorithms to improve accuracy and reliability. By focusing on children under 12, the study aims to facilitate early prediction and intervention, potentially enhancing health outcomes. The proposed model can assist healthcare professionals in early diagnosis and personalized treatment planning for pediatric asthma patients. The performance of these classification algorithms was assessed using K-fold cross-validation, with results indicating that KNN had the lowest accuracy score of 0.63, while Random Forest achieved the highest accuracy of 0.93.

Gunawardana et al. utilized a dataset of 6665 participants from the Sri Lanka Health and Ageing Study and applied thirteen machine learning algorithms, including Logistic Regression, Support Vector Machine, Decision Tree, Random Forest, Naïve Bayes, K-Nearest Neighbors, Gradient Boost, XGBoost, AdaBoost, CatBoost, LightGBM, Multi-Layer Perceptron, and Probabilistic Neural Network, for asthma prediction [12]. Their findings demonstrated that a hybrid model combining Logistic Regression and LightGBM outperformed other models, achieving an AUC of 0.9062 and a sensitivity of 79.85%.

Based on the literature reviewed, the application of AI techniques and acoustic sensing in asthma detection has expanded significantly in recent years. Additionally, Ozsahin et al. investigated the use of multiple machine learning models—including Multi-Linear Regression (MLR), Artificial Neural Networks (ANNs), Adaptive Neuro-Fuzzy Inference Systems (ANFISs), and the Random Forest classifier—in conjunction with peripheral blood smears to develop deep learning frameworks for malaria prediction [13]. Their dataset comprised 2207 patient samples, and the results demonstrated the superior performance of the ANN model.

However, the interpretability of classification results remains a challenge for clinical evaluation. Therefore, this study incorporates feature importance analysis and overall average spectrum techniques to enhance the transparency of ML model results. A summary of related studies is presented in Table 1.

## 3. Proposed AI Prediction System

**Figure 1:**
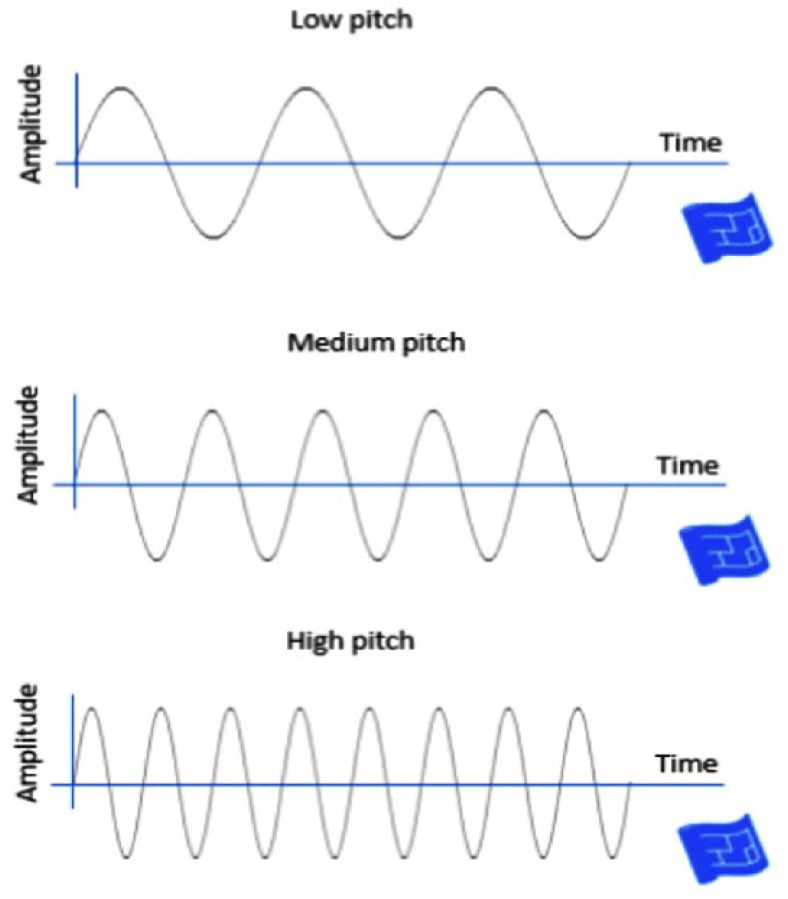
Three sampled pitch speech materials used in this study as acoustic input for classification. Sound waves representing low, medium, and high pitch. As pitch increases, the frequency of the waveform increases while amplitude remains constant, illustrating how pitch affects wave frequency.

**Figure 2:**
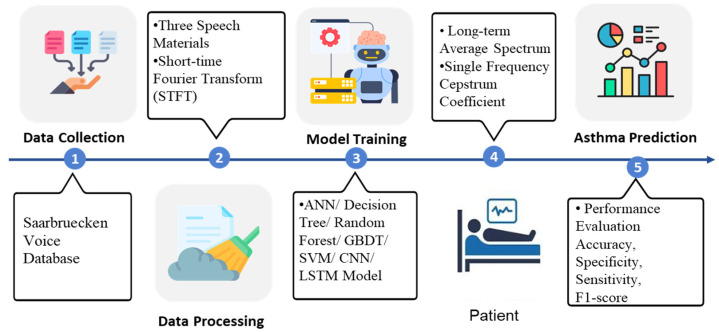
The system architecture, including data source, pre-processing methods, ML models, and performance evaluation.

The asthma detection procedure in this study is separated into six parts: First, three types of speech materials, namely high pitch, normal pitch, and low pitch of [i, a, u] phonemes were obtained from the database. The sampled pitch is shown in Figure 1. Second, feature selection, data cleaning, and data transformation were conducted. Third, the data were input into the machine learning models. Fourth, the features were extracted using Long-term Average Spectrum (LTAS) and Single Frequency Cepstrum Coefficient (SFCC) tools. Fifth, the prediction performance was expressed as accuracy, specificity, sensitivity, and F1-score using the Confusion matrix and being compared. The system architecture is illustrated in Figure 2, and its detailed operations are described in the following sections.

### 3.1. Data Processing

Voice is a critical human physiological characteristic which carries important information. A tiny change in the vocal structure would result in a significant impact on the acoustic feature [14]. In recent years, the application acoustic recognition in the medical field has grown tremendously. Various respiratory acoustic signal types could be analyzed, with cough and wheeze and speech characteristics such as pitch and nasal sound being included [15]. The high diagnosis accuracy for many diseases could provide medical clinician advice. The voice samples that represent Asthma characteristics were obtained from the voice pathological database Saarbruecken Voice Database (SVD, https://www.spsc.tugraz.at/databases-and-tools/saarbruecken-voice-database.html, accessed on 12 December 2024).

These voice dataset sources encompass an extensive collection of voice signals derived from both healthy individuals and asthma patients worldwide. A total of 1500 samples [female 620, male 880] of asthma and healthy patients were analyzed in this study. The expression of human voice and the consistence of articulation can be affected by tissue inflammation and swelling as the velopharyngeal system is abnormal. This study utilized [i, a, u] pronunciation of three consisting of different types of pitch sound. Commonly, low-pitch materials are defined as voice frequency less than 70 Hz, normal pitch as voice frequency around 180 Hz, while a high pitch is above 200 Hz. The obtained acoustic signal was first transformed into frequency spectrum using short-time Fourier transform (STFT) as shown in Figure 1, which retains the characteristic of time domain after transformation [16]. Feature extractions were processed using Long-Term Average Spectrum (LTAS) and Mel-Frequency Cepstral Coefficients (MFCCs) techniques and then fed to seven machine learning models for further classification [17].

The Short Fast Fourier Transform (SFFT) is used to convert voice frames from the time domain to the frequency domain, focusing on time-varying signals. Unlike the standard Fourier Transform, SFFT efficiently preserves time-domain characteristics. The Long-Term Average Spectrum (LTAS) is a common technique for transforming sound signals into frequency spectrums, providing a feature set for classification. LTAS highlights consistent energy distribution across speech, reducing the prominence of individual formants [16]. Frequency bins ranging from 20 to 5000 Hz are extracted for machine learning input. The tools used for this process include Python with the Librosa library for computing STFT and MFCC, and Praat for LTAS analysis (https://github.com/librosa/librosa/tree/main/librosa, accessed on 12 December 2024; https://github.com/praat/praat, accessed on 12 December 2024). In Python, Librosa enables audio loading and feature extraction with just a few lines of code. Averaging across the time dimension produces fixed-size feature vectors suitable for machine learning applications. For this study, a laptop with 16 GB of RAM is sufficient to perform all processing tasks.

**Figure 3:**
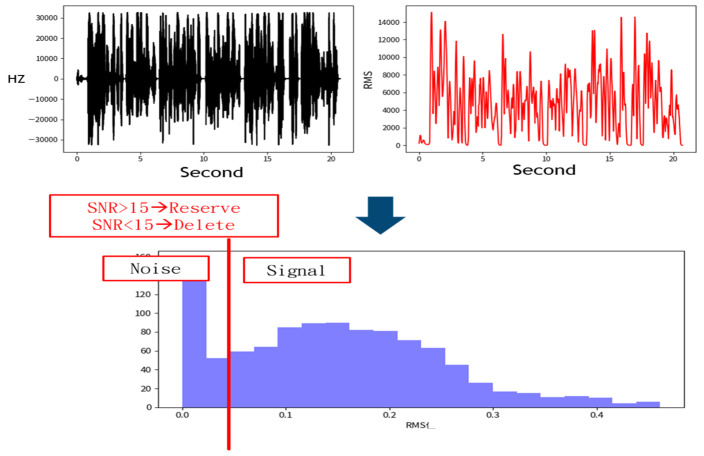
Sampling data processing method. Acoustic signals with a Signal-to-Noise Ratio (SNR) lower than 15 were discarded, while those with an SNR of 15 or higher were retained.

The denoising process removes signals with a low Signal-to-Noise Ratio (SNR), as shown in Figure 3 [16]. This figure illustrates how signals are distinguished from noise based on the SNR. The top-left plot shows frequency variations over time, depicting a fluctuating waveform that likely contains both signal and noise. The top-right plot represents signal intensity using Root Mean Square (RMS), showing significant variations. The middle annotation states “SNR > 15→Reserve” and “SNR < 15→Delete”, indicating that data with an SNR below 15 are classified as noise and filtered out. The bottom histogram displays the RMS distribution, with a red vertical line marking the SNR = 15 threshold. The left side of the histogram represents low RMS values corresponding to low SNR, categorized as noise, while the right side represents higher RMS values corresponding to high SNR, retained as useful signals. The primary objective of this process is to filter out noise using the SNR threshold and retain high-quality signal data.

### 3.2. Feature Processing

For clearly identifying the acoustic feature, methods based on complete ensemble empirical mode decomposition with adaptive noise (CEEMDAN) and optimal wavelet were the used approach for sound denoising [18]. First, the CEEMDAN algorithm was used to decompose the noisy sound signal to obtain several Intrinsic Mode Functions (IMFs) with different scales; then, the information of IMF components was processed according to the characteristics of autocorrelation function. The noise including IMF components was removed. The aliased IMF components were further subjected to optimal wavelet denoising. Finally, the processed IMF component and the remaining IMF component were reconstructed to provide the denoised sound signal. For further signal clearance, TQWT was used to decompose the noisy chaotic signal [19]. The sub-band of TQWT could be accurately divided into signal sub-band and noise sub-band according to the maximum wavelet entropy theory and energy threshold rule. The standard deviation of the singular value subset was used to determine the effective reconstruction order to improve the noise suppression effect. TQWT reconstruction was finally performed on the preliminarily denoised signal to obtain a clear acoustic signal for further classification.

The data features, including different types of pitch sounds and basic personal data, are listed in Table 2. Seven categories are identified based on these features. In this study, the feature importance validation tool was utilized as part of an explainable AI analysis. Feature importance (FI) is used to interpret the machine learning model y = f(x), which establishes the relationship between explanatory variables or features (x) and objective variables (y) [20]. Explainability in statistics and machine learning encompasses various aspects, including quantifying the importance of different features.

The overall average spectrum was obtained through averaging the long-term average spectrum of healthy and asthma groups. The average and standard deviation value were calculated to acquire a frequency spectrum range. Finally, the result was statistically analyzed to verify whether each frequency range shows significance. And the frequency ranges which showed significance were identified. The outcome was then compared with the feature importance result to verify their accordance. The effectiveness of the ML model was then verified.

### 3.3. Model Design

**Figure 4:**
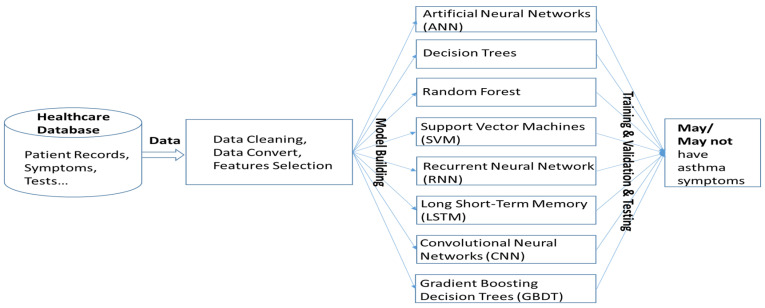
System architecture for asthma symptom prediction using machine learning. Patient data undergo cleaning, conversion, and feature selection before being used to train various models to determine the presence of asthma symptoms.

The machine learning algorithms utilized in this study represent a diverse set of models, each with unique strengths in handling structured and unstructured data [21,22]. Artificial neural networks (ANNs) are inspired by biological neurons and consist of multiple interconnected layers that learn complex patterns through backpropagation. Decision Trees are hierarchical models that split data based on feature conditions, offering high interpretability but being prone to overfitting. Random Forest, an ensemble learning method, improves upon Decision Trees by training multiple trees on random subsets of data and aggregating their predictions to enhance accuracy and reduce variance. Gradient Boosting Decision Trees (GBDTs), another ensemble technique, builds trees sequentially, where each new tree corrects the errors of the previous ones, leading to strong predictive performance. Support Vector Machines (SVMs) classify data by finding the optimal hyperplane that maximizes the margin between different classes, making them effective for high-dimensional datasets. Convolutional Neural Networks (CNNs) excel in processing spatial data, particularly images, by leveraging convolutional layers to extract hierarchical features. In this study, we used AlexNet as the CNN model to analyze asthma-related voice recordings from the Saarbruecken Voice Database. Sustained vowel sounds were converted into MFCC feature maps and resized to fit AlexNet’s input. Lastly, Long Short-Term Memory (LSTM) networks, a type of recurrent neural network (RNN), were designed to capture long-term dependencies in sequential data, making them well-suited for time-series analysis. By leveraging these diverse algorithms, this study aims to identify the most effective approach for predicting asthma based on the given dataset. The system operation is illustrated in Figure 4.

## 4. Performance Analysis

To evaluate the performance of machine learning models, the most commonly used parameters include accuracy (ACC), sensitivity (SEN), specificity (SPE), and F1-score. A total of 1500 samples of three types of speech materials—high-pitch, normal-pitch, and low-pitch recitations of the phonemes [i, a, u]—were used, sourced from the Saarbruecken Voice Database. To prevent overfitting, 80% of the dataset was allocated for model training, while the remaining 20% was used for testing.

**Figure 5:**
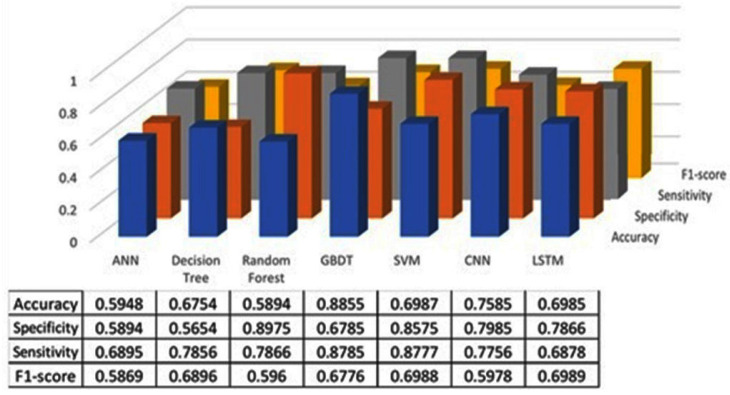
Classification results for high-pitch group.

**Figure 6:**
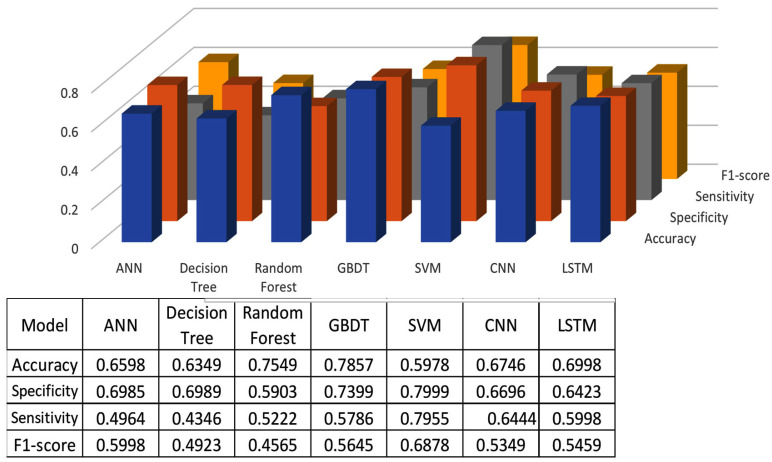
Classification results for normal-pitch group.

**Figure 7:**
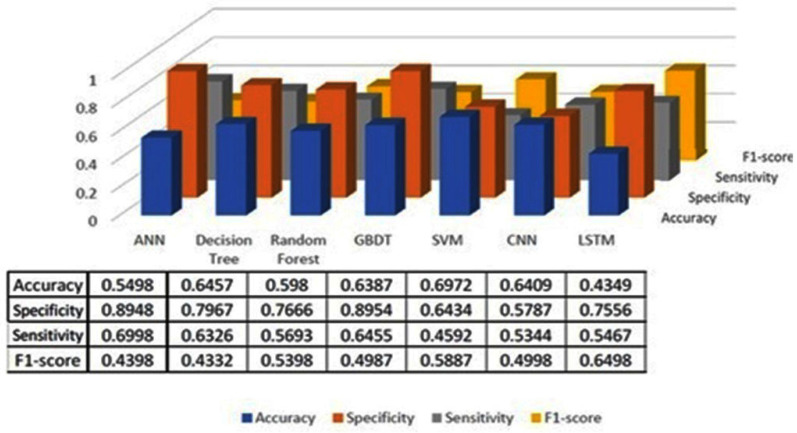
Classification results for low-pitch group.

The classification results using seven machine learning models are summarized in Figure 5, Figure 6 and Figure 7. The highest accuracy was achieved in the following cases: high-pitch passages with the Decision Tree model (Accuracy: 98.66%), normal-pitch passages with the LSTM model (Accuracy: 76.94%), and low-pitch passages with the LSTM model (Accuracy: 85.64%).

In terms of specificity, the best-performing models were high-pitch passages with the LSTM model (Specificity: 0.85), normal-pitch passages with the SVM model (Specificity: 0.76), and low-pitch passages with the Decision Tree model (Specificity: 0.76).

For sensitivity, the highest values were observed in high-pitch passages with the Random Forest model (Sensitivity: 0.85), normal-pitch passages with the CNN model (Sensitivity: 0.85), and low-pitch passages with the ANN model (Sensitivity: 0.69).

Regarding the F1-score, the best performance was obtained with high-pitch passages using the LSTM model (F1-score: 0.75), normal-pitch passages using the SVM model (F1-score: 0.76), and low-pitch passages using the CNN model (F1-score: 0.86).

The reason for different models achieving higher performance across pitch variations is that the feature extraction process prioritizes different aspects of the data depending on pitch characteristics. Different machine learning models leverage distinct features for classification, leading to variations in performance when applied to different types of sound materials.

**Figure 8:**
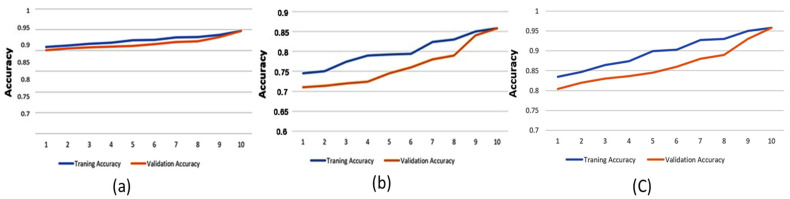
Prediction accuracy of the Decision Tree model using (**a**) high-pitch speech material, (**b**) normal-pitch speech material, and (**c**) low-pitch speech material.

Although machine learning models can provide high decision-making accuracy, some lack transparency and interpretability in their predictions. This makes it challenging for doctors to evaluate the results effectively. One approach to enhancing explainability is quantifying feature importance. In this study, we used Shapley values, a widely adopted method for defining variable importance, to assess the significance of different features. The overall average spectrum was obtained by averaging the Long-Term Average Spectrum (LTAS) of the healthy and asthma groups. The mean and standard deviation values were calculated to determine the frequency spectrum range. A statistical analysis was then conducted to identify frequency ranges with significant differences. The identified significant frequency ranges were compared with feature importance values to assess their consistency. Figure 8 illustrates the prediction accuracy of the Decision Tree model using high-pitch, normal-pitch, and low-pitch speech materials, respectively. The *x*-axis represents the number of training/validation epochs.

## 5. Discussion

Asthma is a common chronic respiratory immunological disorder, with the primary diagnostic method involving the measurement of neutralized antibody levels in blood serum. However, the invasiveness and time-consuming nature of this method limit its clinical efficiency. In recent years, the integration of AI techniques into asthma detection has led to significant advancements. This study uses the changes in voice caused by laryngeal swelling due to asthma to develop an acoustic signal-based prediction system. A total of 1500 speech samples were collected using three types of speech materials: high-pitch, normal-pitch, and low-pitch recitations of the phonemes [i, a, u]. The acoustic signals from these recitations were transformed into frequency spectra, from which Long-Term Average Spectrum (LTAS) and Mel-Frequency Cepstral Coefficients (MFCCs) were extracted as features. These features were then used to train seven supervised machine learning models: Decision Tree, Random Forest, Gradient Boosting (GBDT), Support Vector Machine (SVM), Artificial Neural Network (ANN), Convolutional Neural Network (CNN), and Long Short-Term Memory (LSTM). The models were implemented using the scikit-learn package (version 1.1.2) in Python 3.10.12 for asthma identification.

To prevent overfitting, 80% of the dataset was used for training, with the remaining 20% allocated for testing. The results show that the GBDT model, using high-pitch phonemes, achieved the highest accuracy of 88.6%. Additionally, feature importance validation revealed a significant difference between the healthy and asthma groups in the frequency range of 450–500 Hz, which aligns with the overall average spectrum analysis. This suggests that this frequency range plays a critical role in acoustically analyzing asthma, matching clinical otolaryngological theory. This could be due to resonance changes caused by nasal obstruction during asthma development. Moreover, the results show that prediction accuracy improved with increasing epochs, indicating that the training process in this study was effective. Furthermore, the reason for different models achieving higher performance across pitch variations might be that the feature extraction process prioritizes different aspects of the data depending on pitch characteristics. Different machine learning models utilize distinct features for classification, leading to variations in performance when applied to different types of speech materials.

Due to differences in the adopted dataset and the learning parameters set, the performance of the proposed methods cannot be directly compared with the existing methods listed in Refs. [10,11,12,13]. However, the GBDT model, when combined with high-pitch phoneme speech data, proves to be highly effective in identifying asthma. The accuracy of the proposed GBDT model reaches the performance level of the existing methods. This approach requires minimal instrumentation, presenting a promising solution for the early detection and management of respiratory diseases in the future.

## 6. Conclusions

This study introduces an AI-driven acoustic signal analysis technique to assist in clinical asthma detection. Three types of speech materials were analyzed to extract acoustic features, with Long-Term Average Spectrum (LTAS) and Single-Frequency Cepstral Coefficients (SFCCs) serving as input features for machine learning models. Seven machine learning algorithms—Decision Tree, Random Forest, GBDT, SVM, ANN, CNN, and LSTM—were employed for classification. The results show that the Decision Tree model, combined with high-pitch speech materials, achieved the highest accuracy of 98.7%. To improve result transparency, feature importance analysis and overall average spectrum visualization were incorporated to enhance clinical interpretability. In summary, this study presents a non-invasive and highly efficient asthma prediction method by integrating AI and acoustic sensing techniques.

## Figures and Tables

**Table 1:** Related articles of AI application in asthma prediction.

Ref.	Dataset	AI Model	Feature	Performance
[10]	3000 for training records and 500 for testing records	SVM, KNN, and CNN	Patient Id, Date, Temperature, Humidity, Atmospheric pressure, and fine dust	Accuracy of 96.7%
[11]	698 ACD questionnaires, 340 FeNO measurements, and 1348 spirometry measurements	SVM, Random Forests, and AdaBoost	ACD score and the daily assessment of the fractional exhaled nitric oxide (FeNO), controller usage, reliever usage, and forced expiratory volume in 1 s (FEV1)	Accuracy of 0.79 compared to Random Forests (0.84) and AdaBoost (0.84)
[12]	152 samples, 24 routine blood markers	Decision Trees, Random Forests, Support Vector Machines (SVMs), Neural Networks, and Bayesian Networks	White blood cell, Neutrophil, Lymphocyte, Monocyte, Eosinophil, Basophil	Accuracy: FWAdaBoost (0.61), MLFE (0.60), SVR (0.64), SVM (0.69) and ERM (0.68)
[13]	2207 Malaria patients	ANFIS, Random Forests, MLR, and ANN	Fever Symptom, Temperature, Rapid Diagnostic Test (RDT), White Blood Cell Count (WBC) Red Blood Cell Count (RBC), Hemoglobin Level	Accuracy: ANFIS (97%), MLR (92%), and Random Forests (68%)

**Table 2:** Seven types of categories.

Category	1	2	3	4	5	6	7
Pitch Sound	[i, a, u] pronunciation of three consisting of different types of pitch sound						
Age Group	Children	Young	Young	Middle	Middle	Adult	Adult
Age	7–14	15–24	15–24	25–54	25–54	55–80	55–80
Gender	m/f	f	m	f	m	f	m

## Data Availability

The original contributions presented in this study are included in the article. Further inquiries can be directed to the corresponding author(s).
